# Tyrosinase-Based Biosensor—A New Tool for Chlorogenic Acid Detection in Nutraceutical Formulations

**DOI:** 10.3390/ma15093221

**Published:** 2022-04-29

**Authors:** Irina Georgiana Munteanu, Constantin Apetrei

**Affiliations:** Department of Chemistry, Physics and Environment, Faculty of Sciences and Environment, Dunărea de Jos University of Galaţi, 47 Domneasca Street, 800008 Galaţi, Romania; georgiana.munteanu@ugal.ro

**Keywords:** chlorogenic acid, biosensor, manganese phthalocyanine, tyrosinase

## Abstract

The purpose of our research was to develop a new enzymatic biosensor, GPH-MnPc-Tyr/SPE, using as a support screen-printed carbon electrode (SPE) modified with graphene, manganese phthalocyanine, and tyrosinase, with the aim of developing sensitive detection of chlorogenic acid (CGA). To immobilise tyrosinase on the sensor surface, crosslinking with the glutaraldehyde technique was used, thus increasing the enzyme bioactivity on this electrode. The modified electrode has a great catalytic effect on the electrochemical redox of chlorogenic acid, compared to the simple, unmodified SPE. The peak current response of the biosensor for CGA was linear in the range of 0.1–10.48 μM, obtaining a calibration curve using cyclic voltammetry (CV) and square-wave voltammetry (SWV). Subsequently, the detection limit (LOD) and the quantification limit (LOQ) were determined, obtaining low values, i.e., LOD = 1.40 × 10^−6^ M; LOQ = 4.69 × 10^−6^ M by cyclic voltammetry and LOD = 2.32 × 10^−7^ M; LOQ = 7.74 × 10^−7^ M, by square-wave voltammetry (SWV). These results demonstrate that the method is suitable for the detection of CGA in nutraceutical formulations. Therefore, GPH-MnPc-Tyr/SPE was used for the quantitative determination of CGA in three products, by means of cyclic voltammetry. The Folin–Ciocalteu spectrophotometric assay was used for the validation of the results, obtaining a good correlation between the voltammetric method and the spectrophotometric one, at a confidence level of 95%. Moreover, by means of the DPPH method, the antioxidant activity of the compound was determined, thus demonstrating the antioxidant effect of CGA in all nutraceuticals studied.

## 1. Introduction

Phenolic compounds are considered to be substances beneficial for human health through exercising various biological effects, such as scavenging free radicals, modulating enzymatic activity, metal chelation, and the modification of signal transduction pathways [1]. Epidemiological studies have demonstrated the relationship between the intake of foods containing a large amount of phytochemicals and minimising the risk of developing some diseases, such as cancer, coronary diseases, and osteoporosis, and the results of these studies have promoted interest in consuming phenolic compounds from various sources [2].

Phenolic compounds are mainly classified into phenolic and flavonoid categories. An important class within the category of phenolic acids is represented by hydroxycinnamic acids, and chlorogenic acid (CGA) is their main compound [3]. Chemically, chlorogenic acid is the ester of caffeic acid and quinic acid [4].

CGA is widely present in many vegetable products, such as apples [5], potatoes [6], coffee beans [7], grapes [8], plums [9], honeysuckle [10], wormwood [11], cabbage [12] and kiwi [13]. It was noticed that CGA has antioxidant [14], anti-inflammatory [14], anti-hypertensive [15], anti-diabetic [16], anti-neurodegenerative [17] and antitumoral properties [18]. Moreover, most research on the benefits of CGA for human health was carried out in relation to afflictions regarding metabolic disorders, represented by a cluster of conditions that occur together, increasing the risk of heart and blood vessel diseases, stroke, type 2 diabetes, and mortality, in general [19].

Taking into account the biological importance of CGA, detecting the latter in various pharmaceutical or food products has become a necessity. Over the years, more methods for determining CGA and its derivatives in coffee beans, as well as in other vegetable source products, have been developed. Among them, we list the most often used, namely high-performance liquid chromatography (HPLC) [20], capillary electrophoresis [21], micellar electrokinetic chromatography [22], and chemiluminescence [23]. Although the methods described were efficient to quantify CGA and its derivatives, they have certain disadvantages in connection with the high costs of the instruments necessary for employing these methods, and the fact that they are laborious. Although the UV-Vis spectrophotometric method, which is employed in the detection and the quantification of CGA in real samples has some advantages, such as ease of accessibility, quick response, and low costs, it is used with difficulty because of the spectral overlap with caffeine contained in coffee beans [24]. 

Electroanalytical methods, particularly the voltammetric ones, such as (cyclic voltammetry (CV), square-wave voltammetry (SWV), and differential pulse voltammetry (DPV)), satisfy many of the shortcomings of spectral methods and are often used to determine electrocatalytic activity and to investigate the mechanism of electro-oxidation of several compounds, namely p-coumaric acid [25], rutin [26], melatonin [27] and chlorogenic acid [28]. These experimental studies used various electrodes, such as the SPE modified with multi-walled carbon nanotubes (MWCNTs/SPE) [24], titanium dioxide/metal-organic nanocomposite structures (UiO-66-NH_2_/TiO_2_) [29], carbon paste electrodes modified with highly defective mesoporous carbon (DMC) [30], glassy carbon electrodes modified with nanocomposites based on multi-walled carbon nanotubes (MWCNTs), copper oxide nanoparticles (CuONPs) and lignin (LGN) (LGN-MWCNTs-CuONPs-GCE) [31].

Developing an electrochemical biosensor can be adequate for detecting CGA, using the advantages related to the recognition properties of an enzyme immobilised on the surface of an electrode. Furthermore, immobilising an enzyme on electrodes modified with various nanomaterials contributes to exploring alternative and advantageous methods of manufacturing biosensors [32]. Nanomaterials and nanocomposites are extremely adequate as matrices for immobilising enzymes, ensuring a stable surface, good biocatalytic activity, and excellent conductivity [33]. An enzyme frequently used to electrochemically determine hydroxycinnamic acids [34] is tyrosinase—a monooxygenase that catalyses two types of reactions: monophenol hydroxylation at o-diphenols, and o-diphenol oxidation at o-quinone—both using molecular oxygen as the co-substratum [35]. The synergic combination of nanomaterials and tyrosinase contributes to increasing the performance of electrochemical biosensors, being useful in detecting CGA as well. Moreover, using electron transfer mediators that possess electrocatalytic characteristics, including metalphthalocyanines, can increase the electron transfer rate and shift the potential towards lower values [36], thus improving biosensor sensitivity and selectivity [37].

The purpose of the present study is to describe and assess the electrochemical behaviour of an enzymatic biosensor containing graphene, manganese phthalocyanine, and tyrosinase (GPH-MnPc-Tyr/SPE) in determining CGA through various voltammetric techniques. Furthermore, the electroanalytical method used to quantify CGA in three different nutraceutical products will be validated through the Folin–Ciocalteu method. In addition, this study has focused on determining the capacity of reducing the DPPH radical of the three nutraceutical products mentioned.

## 2. Materials and Methods

### 2.1. Reagents and Solutions

To obtain the enzymatic biosensor, a graphene-based SPE (GPH/SPE), purchased from Metrohm DropSens (Oviedo, Spain), was later adjusted in the laboratory. Thus, during the first phase, the modification with manganese phthalocyanine (Fluka) was achieved by pouring an MnPc 10^−5^ M solution in chloroform (Aldrich), obtaining GPH-MnPc/SPE. The second stage consisted of modifying GPH-MnPc/SPE through immobilising the tyrosinase enzyme (Tyr), followed by crosslinking, thus obtaining the GPH-MnPc-Tyr/SPE biosensor.

Tyrosinase from mushrooms (7164 U/mg) was purchased from Sigma-Aldrich and was used without supplementary purification. To immobilise the enzyme, a solution obtained from 3.50 mg/mL tyrosinase in a 10^−1^ M (pH = 7.0) phosphate buffer solution (PBS) was used.

In order to obtain the PBS 10^−1^ M solution, sodium diphosphate and phosphoric acid—purchased from Sigma-Aldrich (Saint Louis, MO, USA)—were used. The NaH_2_PO_4_ solution was calculated, weighed, and dissolved in ultrapure water obtained from a Milli-Q (Millipore, Bedford, MA, USA) system. Adjusting the pH to the value of 7.0 was attained by adding phosphoric acid—the measuring of the pH being carried out with the aid of a pH meter from WTW instruments, Weilheim, Germany.

The quantity of MnPc was added to chloroform so as to obtain a concentration of 10^−5^ M to be used in modifying the GPH/SPE electrode.

The chlorogenic acid was purchased from Sigma-Aldrich, being analytically pure. The stock solution had a concentration of 10^−3^ M chlorogenic acid, using the 10^−1^ M pH 7.0 PBS solution as a supporting electrolyte.

The Folin–Ciocalteu reactive (Sigma-Aldrich, St. Louis, MO, USA) and a 15% sodium carbonate solution (Sigma-Aldrich, St. Louis, MO, USA) were used to validate the electroanalytical method through the Folin–Ciocalteu spectrophotometric method. 

The DPPH 0.1 mM stock solution was prepared by weighing a 0.0018 g DPPH reactive (purchased from Sigma-Aldrich) and dissolving 50 mL of 96% ethanol. The solution thus obtained was kept at room temperature, in the dark. The sample solutions were obtained using the same quantities of vegetable products as the ones used in the electrochemical measurements. Volumes of 2 mL of DPPH solution were measured in the cuvette of the spectrophotometer, to which 0.5 mL solutions from each vegetable product were added, later weighing the absorption for each sample at 517 nm [38].

The compounds that were used for the interference studies (ferulic acid, vanillic acid, p-coumaric acid) were purchased from Sigma-Aldrich. The L-ascorbic acid was purchased from Riedel-de Haën (Seelze, Germany).

### 2.2. Electrodes and Devices Used

The electrochemical measurements were carried out with the aid of a conventional system containing three electrodes, namely: the reference electrode—Ag/AgCl (Princeton, Applied Research), a platinum wire as a counter electrode, and working electrodes—GPH/SPE, GPH-MnPc/SPE or GPH-MnPc-Tyr/SPE.

An EG&G 263A model (Princeton Applied Research, Oak Ridge, TN, USA)—controlled by the ECHEM software—potentiostat/galvanostat was used to characterise and optimise the electrode signals, as well as to carry out the electroanalysis of the nutraceutical products. Analytical AS 220/C/2 scales were used to weigh the substances and the Elmasonic (Carl Roth GmbH, Karlsruhe, Germany) ultrasound bath was employed to dissolve the substances.

FTIR spectra were obtained with a Bruker ALPHA FTIR spectrometer (Bruker Optik GmbH, Ettlingen, Germany), using the OPUS (Bruker Optik GmbH, Ettlingen, Germany) software in the range of 4000–500 cm^−1^, in the attenuated total reflectance mode (ATR). The ATR ZnSe crystal was wiped clean with ultrapure water and isopropanol between the measurements. The background was the spectrum obtained in the air (ATR ZnSe crystal).

The surface of the biosensor was analysed with a scanning electron microscope (FlexSEM 1000 II Hitachi, Tokyo, Japan).

### 2.3. Preparation of the GPH-MnPc/SPE Biosensor

To prepare the modified GPH-MnPc/SPE biosensor, several stages were covered. Initially, 10 μL of 10^−5^ M manganese phthalocyanine solution in chloroform was added—sequentially, with pauses for drying, through the drop-and-dry technique—to the surface of the GPH/SPE. Drying was performed at room temperature for 30 min. An Eppendorf micropipette was used to add the manganese phthalocyanine solution. 

### 2.4. Preparation of the GPH-MnPc-Tyr/SPE Biosensor

To prepare the biosensor, GPH-MnPc/SPE was used as the support. Using the drop-and-dry technique, 10 µL were added sequentially, in two stages (5 µL in each), with a 3 h pause for drying in between. The enzyme reticulation was achieved by maintaining the sensor above a container with 5 mL of 2% glutaraldehyde, for 1 min. The glutaraldehyde vapours ensured the immobilisation of tyrosinase on the electrode surface through reticulation. The biosensors were kept at 4 °C until use, for a maximum of 72 h [39]. This immobilisation method produces a three-dimensional matrix in which tyrosinase is tightly stuck with the electrode material; this fact enhances the retention of the biomolecule on the electrode surface and also its electrical communication [40]. 

**Figure 1:**
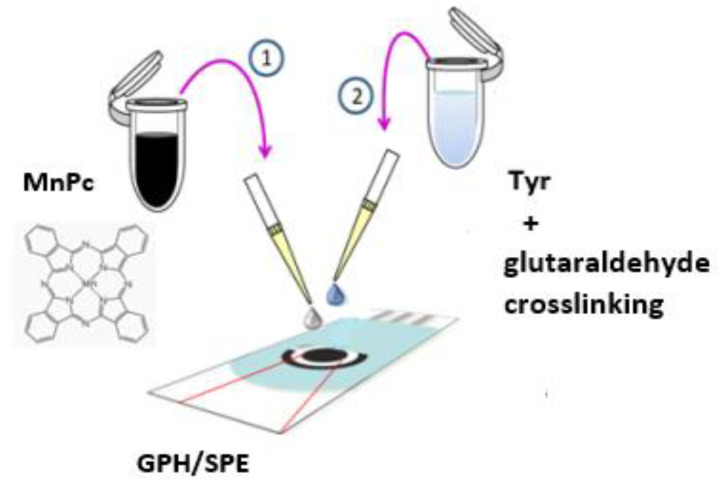
Preparation process of the tyrosinase-based biosensor on the support of an SPE based on graphene modified with manganese phthalocyanine (GPH-MnPc-Tyr/SPE). (1) Addition of MnPc solution; (2) Addition of tyrosinase solution and crosslinking process.

Figure 1 shows the biosensor preparation process.

### 2.5. Methods of Analysis

The most frequent stationary methods employed to analyse phenolic compounds are the voltammetric techniques. The present study has used two electroanalytical methods to highlight the oxidation-reduction processes occurring on the surface of the electrode, as well as to validate the results obtained.

#### 2.5.1. Cyclic Voltammetry

Cyclic voltammetric measurements can be used to determine the antioxidant properties of many active species [41]. These properties are closely related to the chemical structure of the respective species and their redox properties [42]. The technique was used to characterise the working electrodes and to detect CGA, on the one hand in the solution prepared with pure substance and, on the other hand, in the solutions obtained from real samples—represented by nutraceutical products.

#### 2.5.2. Square-Wave Voltammetry

Square-wave voltammetry has a high analysis rate and has proved to be especially sensitive in determining electroactive organic compounds due to the very low non-faradic current [43]. Moreover, the peaks corresponding to the oxidation or reduction of electroactive compounds on the surface of the electrode can be achieved within the same experiment, and the reversibility of the electron transfer can be examined in one scan, the current being measured both during the negative impulses and during the positive ones [44]. The results obtained using the two voltammetric methods described above were compared; supplementary information was extracted. The potential range used was from −0.4 to 0.7 V, the pulse height was 0.10 V, at a frequency of 15 Hz and a pulse potential increment of 7 mV.

### 2.6. Real Samples and Preparation of the Solutions to Be Analysed

The nutraceutical products used in the analysis were purchased from a store specialised in organic products—the pharmaceutical form of all three products being the capsule. The formulations contain a variety of active compounds and excipients, with each prospect mentioning the presence of CGA, the substance of interest for the present study.

Green Coffee Bean 400 mg Jarrow Formulas is a certified product, made by Secom. This product contains green coffee bean extract, being sold as vegetable capsules, which can be administered in vegetarian/vegan diets as well. The chlorogenic acid content is of a minimum 50%, and the compound is obtained through a patented process by Applied Foods Science, Inc., out of pure green coffee (not roasted or processed, ecologically cultivated). It is recommended: to reduce body fat deposits; stimulate the burning of fatty acids; reduce the synthesis, maintain and deposit of triglycerides and cholesterol; adjust the glucose level and reduce complications resulting from its increase in the blood; reduce the absorption of glucose at the intestinal level and improve the sensitivity of cells to insulin; reduce the liberation of glucose and its depositing in the liver as well as to protect cells against oxidative stress. 

Green Coffee Bean Complex (Adams Vision) is a product that is recommended to block fat depositing in the adipose tissue and stimulate circulation. The main components of the product are as follows: green coffee, 350 mg; green tea, 50 mg; anhydrous caffeine, 50 mg; apple cider vinegar, 25 mg; grapefruit, 25 mg; sea algae, 25 mg; acai, 25 mg; African mango, 10 mg; resveratrol, 5 mg. Due to its green coffee content, Green Coffee (Biotech USA) limits the absorption of glucose by the human body. The product formula also includes chrome, which helps to maintain an optimum level of glucose in the blood, and a healthy metabolism.

The voltammetric technique used was CV, in the potential range from −0.4 V to 0.7 V. The content of a capsule of each nutraceutical product was dispersed in 50 mL of PBS (pH = 7.0) solution for the electrochemical analysis. To prepare the real samples for analysis, an ultrasonic bath was used for homogenisation, and the insoluble substances were separated through filtering.

In order to validate the proposed electroanalytical technique, we used the Folin–Ciocalteu spectrophotometric method. The standard solutions were those prepared with pure chlorogenic acid at different concentrations [45]. For this spectrophotometric method, nine standard solutions were used, with final concentrations between 0.28 and 2.53 mg/mL, obtained from the different volumes of the chlorogenic acid stock solution. Subsequently, 2 mL of 15 % Na_2_CO_3_ and 0.5 mL of the Folin–Ciocalteu reagent solution was added, making up to a final volume of 5 mL with ultrapure water. The solutions thus obtained were stored at room temperature for 30 min, then, the absorbances were recorded with a Rayleigh UV/VIS spectrophotometer with a double beam (Beijing Beifen-Ruili, Beijing, China), at 765 nm.

In the case of nutraceutical products, the samples were prepared according to the same model as in the case of the voltammetric analysis. Dilutions from the stock solution were made so that absorbance should be almost half of the absorbance interval obtained for the standard solutions. All the nutraceutical formulations were analysed in triplicate.

### 2.7. Antioxidant Activity (DPPH Method)

The DPPH test is a simple and rapid method, usually used to assess the antioxidant characteristics of vegetable compounds or extracts, particularly those of phenolic compounds [46]. This method is based on the hydrogen atom donation to antioxidants in view of neutralising the DPPH radical (2,2-diphenyl-1-picrylhydrazyl). The reaction is accompanied by a decrease in the intensity of the purple colour of the solution, which contains the DPPH radical, measured at 517 nm; the discoloration of the solution being an indicator of antioxidant efficacy [41].

## 3. Results and Discussion

### 3.1. Preliminary Studies to Characterise the Electrode

In order to highlight the changes in the GPH/SPE after adding manganese phthalocyanine and tyrosinase to the active surface of the two working electrodes, the FTIR spectrophotometric method (standard method) [47] was used and the results are presented in Figure S1.

Several peaks, representative of the presence of tyrosinase, within the 1450–1650 cm^−1^ range, can be noticed [48]. In both cases, the absorption corresponding to the 3000–3700 cm^−1^ range is attributed to the stretching vibration of the hydroxyl groups [49]. The absorption, at approximately 1630 cm^−1^, is attributed to the structural vibration of the graphene, indicating the sp^2^ hybridisation of C atoms [50]. Moreover, the band from 1253 cm^−1^ appears due to the elongation vibrations of the C–N bond, while the band from 1484 cm^−1^ corresponds to the elongation of the C–C bond in the benzenic ring [51]. Moreover, absorption at approximately 2924 cm^−1^ is attributed to the stretching vibration of the methyl groups in the structure of the manganese phthalocyanine [52].

**Figure 2:**
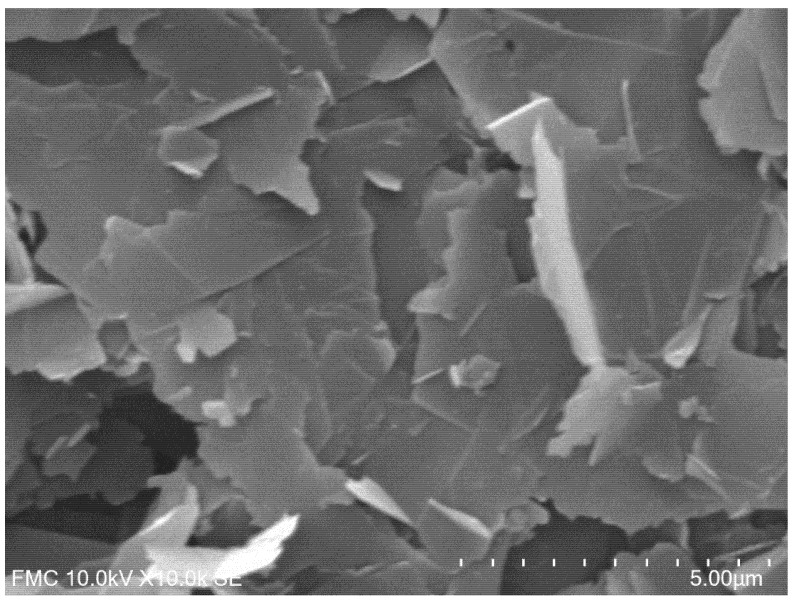
SEM picture of the GPH-MnPc-Tyr/SPE surface.

The morphology of the biosensor surface was characterised by scanning electron microscopy (SEM). Figure 2 shows a scanning electron microscope picture of the surface morphology of the composite nanofilm containing graphene, manganese phthalocyanine, and tyrosinase, where the graphene nanosheets and other components are well evidenced.

To estimate the optimum quantity of tyrosinase necessary to modify the sensor, the influence of the quantity of immobilised enzyme was evaluated in the 160–800 range of the enzyme units (Figure S2). The cathodic current, which corresponds to the ortho-quinone reduction enzymatically formed at catechol, increased with the increase in the enzyme quantity up to 749 U of tyrosinase, followed by a slight decrease. This can be attributed to certain effects, such as the increase in electrical resistance, which makes the electron transfer more difficult, as well as the inaccessibility of the substratum to the active enzyme sites. Therefore, the 749 U enzyme value is optimum for preparing a biosensor with very good sensitivity.

The influence of pH on the enzymatic activity of tyrosinase on detecting catechol in the pH range between 2.0 and 9.0 was preliminarily investigated. Tyrosinase is an enzyme that belongs to the class of oxidoreductases; consequently, the presence of H^+^ ions in the reaction environment represents an important feature, affecting its activity. The activity of the enzyme showed an obvious dependency on the pH, and the current has optimum values for it in the 6.0–7.0 range. Nevertheless, the maximum activity of tyrosinase was noticed at pH = 7.0, which is expressed as the percentage of relative activity in Figure S3. A lower pH value can lead to a more rapid distortion of the enzyme [53]. Moreover, according to the specialised studies, it was noticed that the optimum pH value for detecting phenolic compounds is 7.0 [54]; the modifications in pH influence the protonation process—a constituent part of the redox reaction of these compounds, which implies electron and proton exchange [55]. Therefore, the following experimental analyses employed a PBS 10^−1^ M solution, with pH = 7.0, as a supporting electrolyte. Further preliminary analyses consisted of the evaluation of the electrochemical behaviour of GPH/SPE, GPH-MnPc/SPE, and GPH-MnPc-Tyr/SPE in phosphate buffer solutions (PBS) of 10^−1^ M at pH = 7.0. According to previous studies, the potential range in which a stable signal was obtained was from −0.4 V to 1.3 V [28]. In the case of GPH/SPE, it was noticed that no peaks were highlighted in the potential range studied, which demonstrates the absence of contaminations on the active surface of the electrode that might influence the electrochemical responses, such as graphene high purity.

On immersing the GPH-MnPc/SPE sensor in PBS solution at pH = 7.0, the CVs highlighted a quasi-reversible and irreversible behaviour, which does not change with the scan rate. This can be explained by the fact that manganese phthalocyanine has complex electrochemistry, since the central manganese metal passes through multiple oxidation states, varying from Mn^I^ to Mn^IV^ [56].

The CVs of the two modified electrodes immersed in PBS (pH = 7.0) solution are presented in Figure 2. The aspect of the cyclic voltammograms recorded indicates the electrode signal change during the first cycles. However, the signal was stabilised after three cycles.

**Figure 3:**
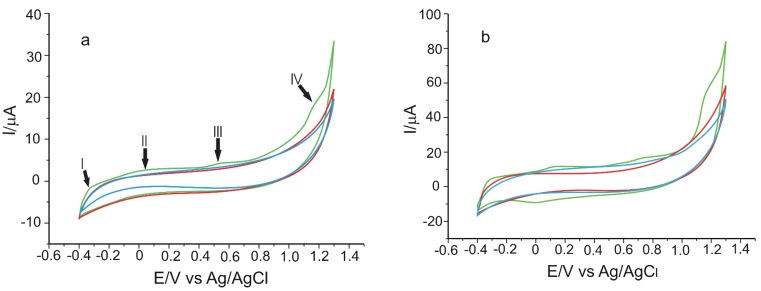
Cyclic voltammograms of GPH-MnPc/SPE (**a**) and GPH-MnPc-Tyr/SPE (**b**) immersed in 10^−1^ M PBS solution, pH = 7.0. Scan rate 0.1 V × s^−1^. Three successive cycles.

Figure 3a shows that the cyclic voltammograms highlighted four peaks at the following values of the potential: −0.237 V (I), +0.220 V (II), +0.657 V (III), and +1.127 V (IV). The two irreversible peaks localised in regions III and IV can be attributed to the oxidation of the metal (Mn(IV)Pc(-2)/Mn(III)Pc(-2) (process III) and to the further oxidation of the aromatic ring (Mn(IV)Pc(-1)/Mn(IV)Pc(-2) (process IV), according to the literature [57,58]. The reversible voltammetric couple situated at +0.220 V corresponds to the Mn(III)Pc(-2)/Mn(II)Pc(-2) (II) redox process, and the one situated at −0.237 V to the Mn(II)Pc(-2)/Mn(I)Pc(-2) (I) redox process [57]. Furthermore, CVs of the biosensor in PBS solution at pH = 7.0 were recorded (Figure 3b). In CVs, the characteristic MnPc peaks can be noticed, and the presence of tyrosinase can be highlighted through the more accentuated anodic peak, at a potential of 1.176 V, as well as through the cathodic peak, at a value of 0.022 V. The background current, in the case of the biosensor, is reduced, which can result in increased sensitivity to CGA detection.

In the next stage, the three sensors were immersed in a 10^−3^ M catechol—10^−1^ M PBS solution, at pH = 7.0, recording CVs at 0.1 V × s^−1^ scan rate, using a potential range between −0.4 and +1.3 V. The two pairs of anodic and cathodic peaks, which are highlighted in the cyclic voltammograms of all three sensors, are due to the reversible redox processes of the catechol on the electrode surface. The nature of the materials used to modify the electrode influences the shape and position of the peaks. The graphene nanostructure is compatible with the enzyme and with the manganese phthalocyanine and, as a result, the hybrid bio-nanocomposite material is highly sensitive. 

The main parameters obtained from the CVs of the three sensors, as well as those that can be calculated from these parameters, are shown in Table S1. In all three cases, it is found that the Ipc/Ipa ratio is close to 1 (ideal value), however, the electrode modified with the mediator and enzyme—GPH-MnPc-Tyr/SPE—showed the highest degree of reversibility, with less separation between the anodic and cathodic peaks compared to the other electrodes, and the Ipc/Ipa ratio was greater than 1 (1.27), indicating that the reaction process at the modified electrode is a quasi-reversible one. All three electrodes have similar electrochemical behaviour according to the parameters obtained and can be used successfully in further determinations.

To determine the kinetics of the process, the cyclic voltammograms of the sensors were registered with different scan rates, ranging from 0.1 to 1.0 V × s^−1^. This was useful for estimating the active surface of the electrodes. The 10^−3^ M catechol–10^−1^ M PBS solution of pH = 7.0 was used in this study and the results obtained for all three electrodes are presented in Figure S4**.**

In Figure S4, it may be observed that the currents corresponding to the oxidation-reduction process of catechol increase when the scan rate increases. To determine the limiting factor of the electrochemical oxidation process, linear regression models were described, correlating the Ipa with the scan rate or with the square root of the scan rate. For all three electrodes, very good linearity was obtained between the Ipa and the square root of the scan rate. According to the literature, this linear dependence suggests that the electrochemical process at the working electrode surface is controlled by the diffusion of the electroactive species, with this being the determining stage of kinetics [28]. 

The active areas of the electrodes were calculated with the Randles–Sevcik equation [27] using the linear dependence between Ipa and v^1/2^.

Ipa = 268600 × n^3/2^ × A × D^1/2^ × C × v^1/2^ (1)

where Ipa is the current of the anodic peak (A), n is the number of electrons transferred following the redox process, A is the electrode area (cm^2^), D is the diffusion coefficient (cm^2^·s^−1^), C is the concentration, (mol·cm^−3^), v = scan rate (V·s^−1^). For catechol, the diffusion coefficient is D =1.913 × 10^−6^ cm^2^·s^−1^ [59].

The values obtained for the linear regression equation, the determination coefficient (R^2^), active surface areas, geometrical areas, and the roughness factor in the case of all electrodes studied are given in Table 1.

It was noticed that the highest value of the active area was obtained for GPH-MnPc-Tyr/SPE, followed by GPH-MnPc/SPE and by GPH/SPE. This is due to the modification of the graphene electrode surface with manganese phthalocyanine (used as an electron mediator, which facilitates the electron transfer and has good biocompatibility with the enzyme) and with tyrosinase, an enzyme that ensures selectivity to the detection of phenolic compounds [54]. The results obtained are in accordance with the values of peak currents obtained in the cyclic voltammograms. From these values, it can be concluded that the sensitivity of the sensors increases in the following order: GPH/SPE, GPH-MnPc/SPE, and GPH-MnPc-Tyr/SPE.

### 3.2. Electrochemical Properties of Electrodes in CGA Solution

According to the preliminary studies, the optimal pH value for phenolic compound detection is 7.0, with the peaks obtained at this pH value being clearer and well-defined. A lower value of pH may lead to faster degradation of the tyrosinase enzyme. Therefore, a solution of PBS 10^−1^ M of pH = 7.0 was used as a supporting electrolyte for the following experimental analysis.

The qualitative and the quantitative determination of CGA was carried out through cyclic voltammetry and square-wave voltammetry. These methods are useful in understanding the electrochemical processes which take place on the surface of the electrodes. GPH-MnPc/SPE and GPH-MnPc-Tyr/SPE electrodes were used at this stage of the study and the scan rate was 0.1 V × s^−1^. To obtain a stable response of the electrodes, three cycles were necessary for the optimised potential range (from −0.4 V to 0.7 V).

**Figure 4:**
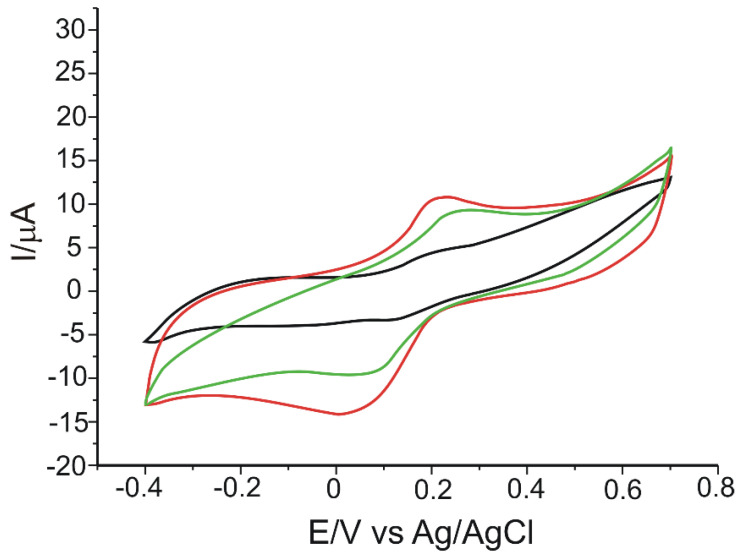
CVs of GPH-MnPc/SPE (black line), GPH-Tyr/SPE (green line), and GPH-MnPc-Tyr/SPE (red line) immersed in 10^−3^ M CGA solution (support electrolyte 10^−1^ M PBS solution). Scan rate 0.1 V × s^−1^.

Figure 4 shows the stable responses of the modified working electrodes at each stage of modification, immersed in CGA 10^−3^ M PBS 10^−1^ M solution (pH = 7.0).

All three voltammograms highlighted an anodic and a cathodic peak of different intensities and potentials, corresponding to the redox processes of CGA at the electrode surface. In the case of the GPH-MnPc-Tyr/SPE biosensor, peaks are the most intense, and the cathodic peak appears at lower potential values as compared to the GPH-Tyr/SPE biosensor, which does not have a mediator in the sensing layer. Therefore, the mediator used, MnPc, facilitates the electron exchange and has a positive electrocatalytic effect, lowering the potential necessary for the reduction of CGA quinonic derivative, enzymatically and/or electrochemically formed [3].

The electrochemical parameters obtained for all the (bio)sensors developed in this study are presented in Table 2.

GPH-MnPc-Tyr/SPE stands out through its lower value of the cathodic peak potential, which suggests that the reduction process needs low activation energy and is related to the presence of Tyr, being a specific property of the enzyme [52]. 

It may be noticed that Epa corresponding to the oxidation of CGA at the GPH-MnPc-Tyr/SPE is higher than the GPH-MnPc/SPE (the oxidation peak potential positively shifted from 0.190 V to 0.214 V), while the cathodic peak potential is lower (the reduction peak potential negatively shifted from 0.117 V to 0.017 V). This shift of the peak towards negative values of the potential demonstrates that the reduction process is greatly influenced by the presence of tyrosinase on the sensing layer [60]. Furthermore, the Ipc/Ipa ratio value >1, in the case of GPH-Tyr/SPE and GPH-MnPc-Tyr/SPE, suggests that the reaction process at the modified electrodes is quasi-reversible. It was also previously demonstrated that the presence of both the mediator and the enzyme increases the active area of the electrode, thus, the background voltammetric response (capacitive current) and sensitivity of MnPc-Tyr—the coated surface is higher than that for the unmodified surface. Therefore, as can be observed from the experimental data (taking into account the values of the parameters E^1/2^ and Ipc/Ipa), it is clearly the greater sensitivity of the biosensor, both in the anodic and cathodic branches, toward CGA detection. Due to the fact that the cathodic peak was more intense compared to the anodic one, subsequent determinations and calculations were reported for its changes. Since the CGA has a 1,2-dihydroxybenzene moiety, the oxidation of this compound should form their respective o-quinone and release two electrons and two protons. The mechanism of chlorogenic acid oxidation on the modified electrode, GPH-MnPc/SPE, is similar to that previously reported [28].

**Figure 5:**
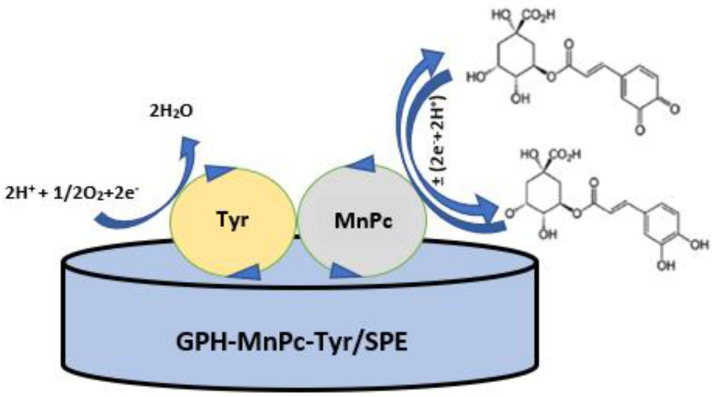
Mechanism of the chlorogenic acid electrochemical detection at the GPH-MnPc-Tyr/SPE surface.

The mechanism of the biosensor developed in this study for the detection of chlorogenic acid is presented in Figure 5.

For the GPH-MnPc-Tyr/SPE biosensor, a redox process was noticed, which includes the exchange of two electrons and two protons in one single stage. The CGA redox process at the surface of the biosensor is biocatalysed by the Tyr. Tyrosinase is an enzyme that has two catalytic sites: one for the hydroxylation of phenols (cresol activity), and one for the oxidation of diphenols up to quinone (catechol activity); both sites being active in the presence of molecular oxygen. Tyrosinase is a metal enzyme that has two copper ions at the level of the active enzymatic site, with each coordinated by means of three histidine waste from the enzymatic polypeptide chain. The oxidation-reduction of tyrosinase is ensured by the reversible transfer of electrons by means of copper ions (${\mathrm{Cu}}^{+}↔{\;\mathrm{Cu}}^{2+})$. Consequently, tyrosinase has the capacity to catalyse the chlorogenic acid oxidation process.

The MnPc facilitates the electron transfer and the effect is mainly observed in the reduction process; the potential is shifted towards a lower potential, and the sensitivity of the biosensor is significantly increased [61]. At a scan rate of 0.1 V × s^−1^, the obtained peaks have reduced intensities and are less obvious due to the influence of the capacitive current. At higher scan rates with higher Faradaic currents, the peaks are better defined [25].

**Figure 6:**
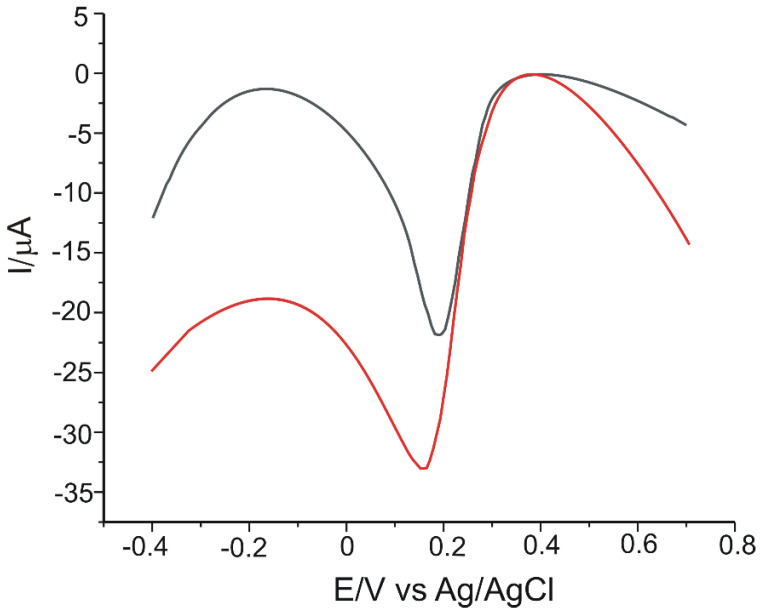
Square-wave voltammograms obtained for GPH-MnPc/SPE (black line) and GPH-MnPc-Tyr/SPE (red line) by immersion in 10^−3^ M CGA solution (10^−1^ M PBS electrolyte pH = 7.0). The potential range was between −0.4 and +0.7 V, pulse height 0.10 V, and a potential increment of 7 mV at a frequency of 15 Hz.

The two electrodes, GPH-MnPc/SPE and GPH-MnPc-Tyr/SPE were used to record the square-wave voltammograms in 10^−3^ M CGA solution (support electrolyte 10^−1^ M PBS of pH = 7.0). The potential range studied was between −0.4 V and +0.7 V, with a pulse height of 0.10 V, an increment of the potential of 7 mV, and a frequency of 15 Hz. Through this method, similar results to those of the cyclic voltammetry were obtained. With both of the electrodes, there was a highlighted intense and better-defined reduction peak and a reduced background current. The square-wave voltammograms are presented in Figure 6.

### 3.3. Influence of Scan Rate on the Voltammetric Response

The next stage consisted of the study of the electrochemical behaviour of the two electrodes in 10^−3^ M CGA solution (the supporting electrolyte was 10^−1^ M PBS of pH 7.0), applying scan rates between 0.1–1.0 V × s^−1^.

**Figure 7:**
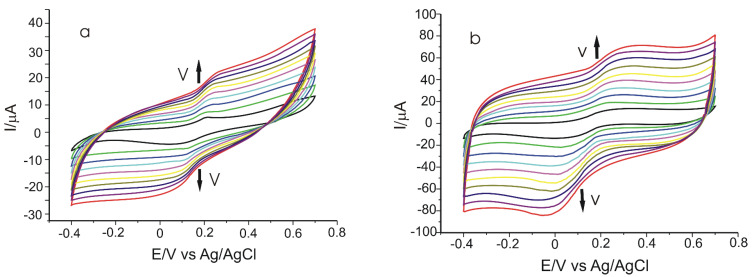
CVs of GPH-MnPc/SPE (**a**) and GPH-MnPc-Tyr/SPE (**b**) recorded at various scan rates within the range 0.1–1.0 V × s^−1^.

Figure 7 presents the CVs of the two modified electrodes recorded for different scan rates.

A progressive increase in the peak intensity with the increase in scan rate was noticed. Taking into account the fact that, in the case of the sensor modified with Tyr enzyme, there is an increase in the cathodic peak current and a shift of the later at more negative values of the potential when the scan rates increase. The dependence between I_pc_ and the scan rate values was studied in order to determine the kinetics limiting stage in the electrochemical detection. 

**Figure 8:**
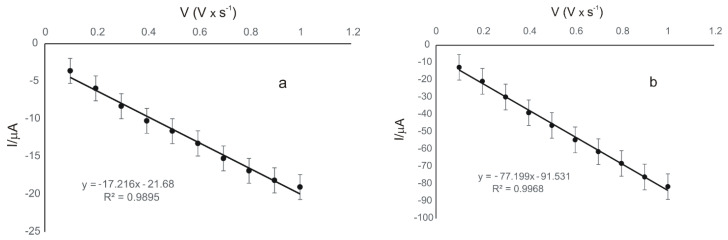
Linear dependence between the cathodic peak currents (Ipc) and the scan rates for GPH-MnPc/SPE (**a**) and GPH-MnPc-Tyr/SPE (**b**).

Figure 8 presents the linear dependence between the cathodic peak currents and the scan rates in the case of the two modified electrodes.

In both cases, there is a linear dependence between the cathodic peak current and the scan rate, which demonstrates that the electrochemical process on the surface of the electrodes is controlled by the absorption of the electroactive species; this being the determining stage of the electrochemical process [24]. 

The coverage degree of the electrode surface with the electroactive species (Γ) was calculated using the equation of the dependence between the current of the cathodic peak and the scan rate, according to the Laviron equation [53].

(2) $$\mathrm{Ip}=\frac{n^{2}F^{2}\Gamma \mathrm{Av}}{4\mathrm{RT}}$$

where Г—concentration of the active species, mol×cm^−2^; Ip—current corresponding to the peak, A; A—surface of the electrode, cm^2^; n—number of electrons transferred during the redox processes; F—Faraday constant, 96,485 C × mol^−1^; R—universal gas constant, 8.314 J × mol^−1^ × K^−1^; T—absolute temperature; K—298 K.

Comparing the results obtained with GPH-MnPc/SPE and with GPH-MnPc-Tyr/SPE, we can say that in both cases the reduction process is controlled by the adsorption; the process is more rapid in the case of the biosensor (comparing the slopes of the two linear fitting equations presented in Table 3). The values of the coverage degree of the electrode surface with the electroactive species (Г) obtained from the Laviron equation are also presented in Table 3.

The Г values obtained for CGA with the two modified electrodes are comparable with those obtained for the other tyrosinase-based biosensors used to detect phenolic compounds and are reported in the literature [61,62,63,64]. Based on these results, it can be affirmed that the biosensor has superior electroanalytical properties in detecting CGA, with Г being one order of magnitude greater for the biosensor, highlighting the importance of Tyr from the active surface. The immobilisation of Tyr, together with GPH and MnPc, promotes increased bioselectivity and conductivity, interfering with the synergistic effect of these materials in biodetection. Since the biosensor has proven to be high-performing in terms of sensitivity and selectivity, it will be used in subsequent quantitative analysis.

### 3.4. Calibration Curve and Limit of Detection 

Based on the higher intensity and lower potential of the cathodic peak, this signal was considered for the quantitative detection of CGA. 

**Figure 9:**
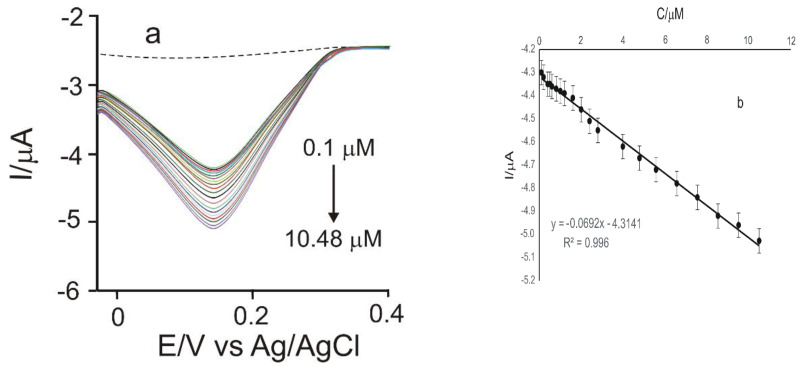
(**a**) Zoom-in of the cyclic voltammograms recorded for GPH-MnPc-Tyr/SPE in the concentration range of 0.1–10.48 µM chlorogenic acid. The dashed line is the biosensor’s response immersed in the electrolyte support solution; (**b**) linear fitting within the range of 0.1–10.48 µM for GPH-MnPc-Tyr/SPE.

The calibration curve was constructed for the sensor modified with MnPc and Tyr, by registering the CVs in CGA 10^−3^ M stock solution, in the concentration range from 0.1 to 678.51 μM (the electrolyte support was PBS 10^−1^ M of pH = 7.0). Variable volumes of CGA solution were used, between 5 µL and 1000 µL, with each stage of volume addition being followed by stirring. The voltammetric responses of the GPH-MnPc-Tyr/SPE immersed in solutions of CGA with different concentrations are shown in Figure 9a. It can be noticed that the intensity of the anodic and cathodic peak increase with the increasing concentration. 

Figure 9b shows the linear dependence between the cathodic peak current and the CGA concentration in the 0.1–10.48 µM range, in the case of GPH-MnPc-Tyr/SPE. For higher concentrations, the electrochemical signal increases more slowly due to the saturation of the active centres on the surface of the working electrode.

LOD and LOQ were calculated using the following equations [65]:

LOD = 3 σ/m; LOQ = 10 σ/m(3)

where σ is the standard deviation (SD) of the electrochemical signal for the blank sample for the potential corresponding to CGA, and m is the slope of the calibration curve.

Table 4 shows the results obtained for LOD and LOQ calculated for the GPH-MnPc-Tyr/SPE biosensor used in the present study.

Due to the presence of tyrosinase, which facilitates the interaction with CGA and confers the biosensor selectivity and sensitivity, the latter has superior performance as compared to the sensor.

**Figure 10:**
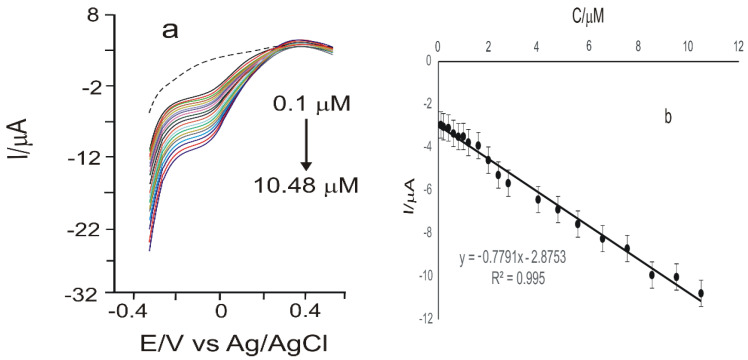
(**a**) Square-wave voltammograms were recorded for GPH-MnPc-Tyr/SPE in the concentration range of 0.1 µM–10.48 µM chlorogenic acid (The potential range is between −0.4 and +0.7 V, pulse height 0.10 V, and a potential increase of 7 mV at a frequency of 15 Hz). The dashed line is the biosensor’s response immersed in the electrolyte support solution; (**b**) linear fitting within the range of 0.1–10.48 µM for GPH-MnPc-Tyr/SPE; y = I (μA); x = c (μM); R^2^—coefficient of determination.

A calibration curve for the same CGA concentration range (0.1 µM–678.51 µM) was also achieved through square-wave voltammetry (Figure 10), for GPH-MnPc-Tyr/SPE. The potential range study was situated between −0.4 and +0.7 V, with an impulse height of 0.10 V, an increase in the impulse potential of 7 mV, and a frequency of 15 Hz. The LOD and LOQ values obtained were 2.32 × 10^−7^ M and 7.74 × 10^−7^ M, respectively, one order of magnitude smaller in comparison with those obtained through cyclic voltammetry.

The values obtained for LOD were compared with those achieved with other sensors and biosensors used to detect CGA, recorded in the literature, as shown in Table 5.

The enzyme-based biosensor described in our study has been successfully used for detecting chlorogenic acid and proved to be a potential and reliable method for the on-site analysis of complex matrices containing chlorogenic acid. The immobilisation of tyrosinase, together with graphene and manganese phthalocyanine, leads to better biosensitivity and conductivity, favouring a synergic effect in biodetection. 

When comparing analytical parameters, such as linearity range and LOD, it can be observed that the biosensor had at least as good performance, and in some cases even better than most (bio) sensors included in Table 5. These results are due to the mediator and the enzyme in the sensitive element of the biosensor and the favourable interaction with chlorogenic acid.

### 3.5. Stability, Reproducibility, and Repeatability of the Biosensor

The stability of the GPH-MnPc-Tyr/SPE biosensor was evaluated through 30 measurements, carried out at regular intervals (1 day) for 1 month, using a CGA 10^−3^ M solution, through cyclic voltammetry. During this period, the sensor was stored in a refrigerator at 4 °C. The results obtained did not highlight important differences between the cathodic currents recorded on different days; the variation coefficients being smaller than 5%, thus confirming that the biosensor is stable and can be used in the electroanalysis.

To verify the reproducibility of the proposed method, we have investigated the response of three biosensors, identically prepared in CGA 10^−3^ M solutions. No differences higher than 2% were noticed between the values of the cathodic current measured for the three biosensors.

The tests for the study of repeatability were carried out in a 50 µM CGA-10^−1^ M PBS solution. The value of the variation coefficient for the cathodic peak, determined in the seven consecutive determinations in the same solution, did not surpass 3%. Between measurements, GPH-MnPc-Tyr/SPE was rinsed with a PBS solution of 10^−1^ M of pH = 7.0. Therefore, the biosensor can be used repeatedly to determine CGA.

### 3.6. Interference Studies

In optimum experimental conditions, the influence of several related compounds, such as L-ascorbic acid, vanillic acid, ferulic acid, and p-coumaric acid, was studied, as these substances could interfere in electroanalysis. The detection technique used was CV. The biosensor signal was determined in the absence and in the presence of the interferent, and the relative response was calculated in percentages. The results obtained show that the cathodic peak corresponding to CGA does not change significantly in the presence of interferents. These are presented in Table 6.

Taking these results into account, it can be affirmed that the GPH-MnPc-Tyr/SPE biosensor has adequate selectivity and precision for determining CGA in real samples.

### 3.7. Quantitative Determination of CGA in Nutraceutical Formulations

For the quantitative analysis of the three pharmaceutical formulations, two methods were employed: cyclic voltammetry (the method developed in this study) and the Folin–Ciocalteu assay.

**Figure 11:**
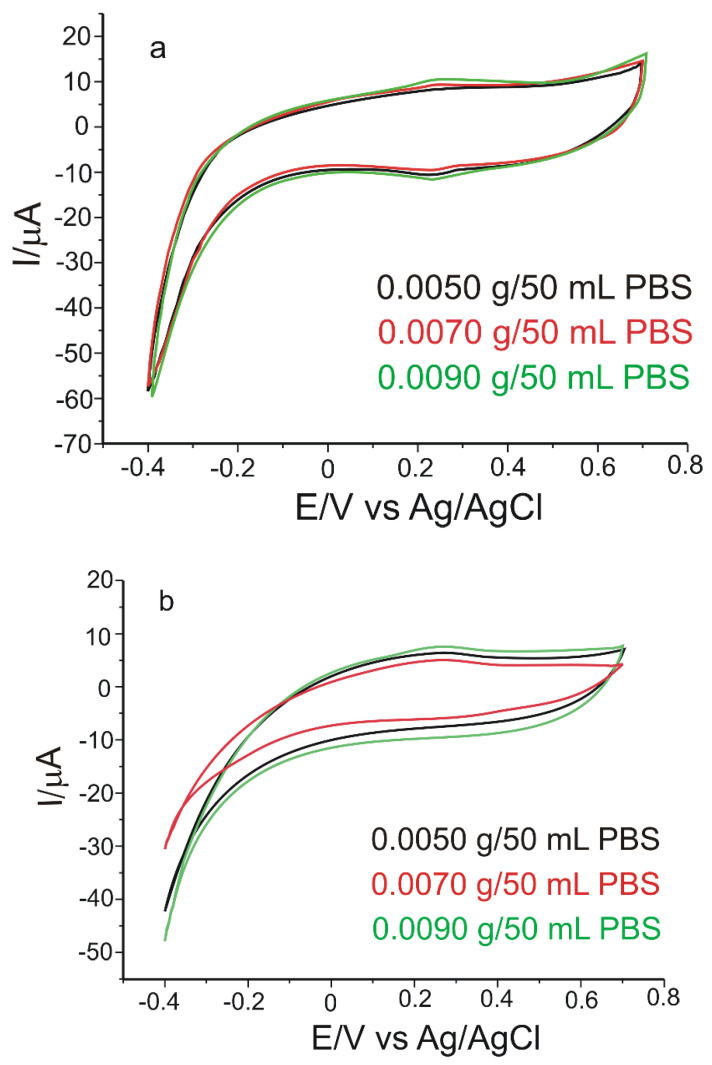

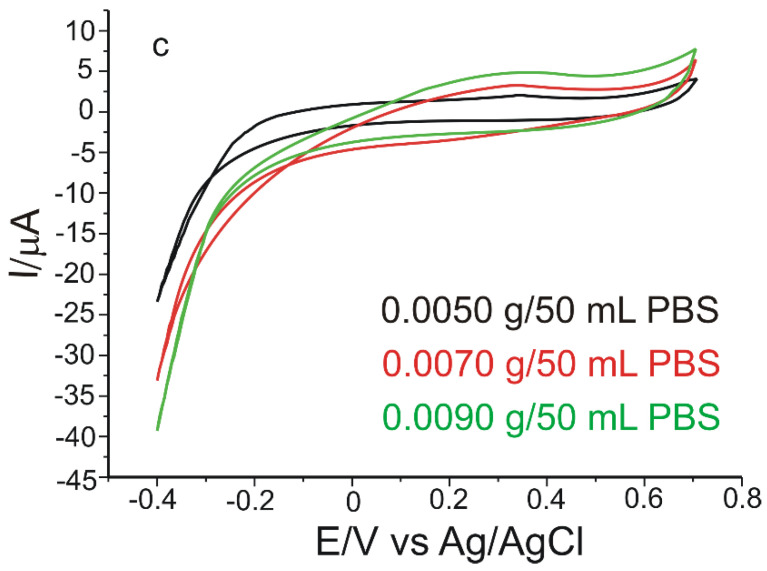
Cyclic voltammograms of GPH-MnPc-Tyr/SPE immersed in a solution with different concentrations prepared from (**a**) Green Coffee Bean Complex (Adams Vision), (**b**) Green Coffee (Biotech USA); (**c**) Green Coffee Bean 400 mg Jarrow Formulas. Scan rate 0.1 V × s^−1^.

With regards to cyclic voltammetry, the measurements were recorded in the potential range situated between −0.4 V and 0.7 V. The kinetics of the redox processes was achieved by recording the cyclic voltammograms with a scan rate of 0.1 V × s^−1^, using three different quantities from each product: 0.0050 g, 0.0070 g and 0.0090 g (Figure 11). 

The three voltammograms indicate the appearance of an anodic peak and a cathodic peak at approximately the same potentials, as in the case of the standard CGA solution. Therefore, GPH-MnPc-Tyr/SPE has better selectivity and sensitivity to detect CGA in pharmaceutical products and nutraceutical products, respectively.

For the Folin–Ciocalteu method, the sample absorbances were recorded at 765 nm after adding the reagents included in the methodology [71]. The calibration curve is achieved from the values of the absorbances obtained for a standard solution of different concentrations. Using the equation of the calibration curve, the CGA concentration in nutraceutical products was determined. 

The quantities of CGA in food supplements were determined based on the calibration equations, which correspond to the two determination methods, namely the spectrophotometric and the voltammetric methods. All the experiments were carried out three times. The results are reported as averages of three repetitions. In calculating the values reported in Table 7, the dilutions and the quantities of food supplements used in the analyses were taken into consideration. The results, expressed as percentage concentrations, are presented in Table 7.

The data presented in Table 7 indicate that the values of CGA obtained through using the two methods are similar, which demonstrates that the voltammetric method is a sensitive, valid, and precise method for detecting the chlorogenic acid in nutraceutical products. The product labels specify that the presence of CGA in each capsule represents a minimum of 50%.

Moreover, this method, which is based on the enzyme-biosensor is very accurate and is also versatile; therefore, it could be used in the routine analysis of quality control of other products, besides those described in this study, and even cosmetic products.

### 3.8. Determination of CGA Antioxidant Activity through DPPH Method

In the first stage of the determination, the DPPH 0.1 mM stock solution was prepared from DPPH reactive and 96% ethanol, kept at room temperature, in the dark. Later, volumes of 0.5 mL from each sample solution (the same as those used in the electrochemical measurements) were measured and added to 2 mL of DPPH solution. Then, measuring the absorbance was minute by minute, for 10 min.

The absorbances were measured by means of a Rayleigh UV2601 UV/Vis double fascicle spectrophotometer (Beijing Beifen-Ruili Analytical Instrument, Beijing, China). The capacity to scavenge DPPH radical was calculated as the percentage of concentration reduction. The lower absorbance of the reaction mixture at the end of the reaction time indicated a higher activity of scavenging DPPH radical. DPPH radical reduction capacity expressed as percentage was calculated according to the following equation [72]:

% DPPH Reduction = 100 × (AD − AE/AD)(4)

where AE is the absorbance of the solution when the pharmaceutical product has been added and AD is the absorbance of the blank DPPH solution.

**Figure 12:**
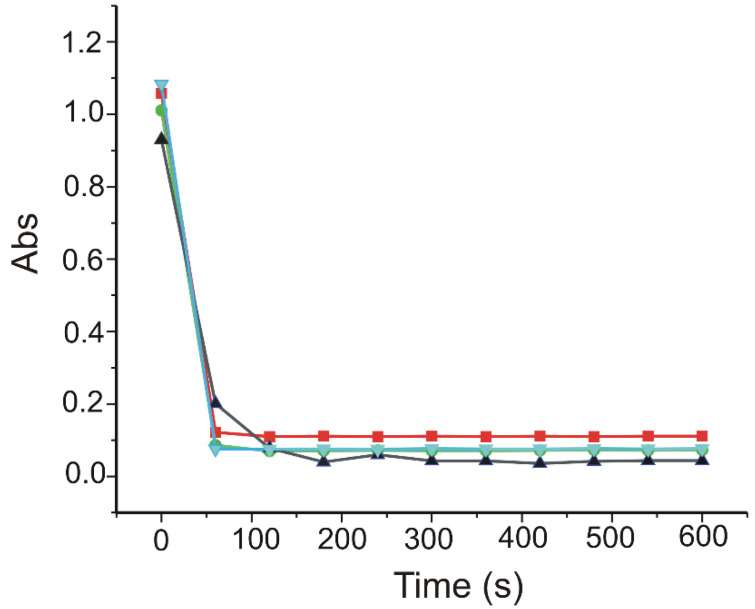
Kinetics scavenging of DPPH radical by CGA (black line); Green Coffee Bean Complex, Adams Vision (red line); Green Coffee, Biotech USA (blue line); and Green Coffee Bean 400 mg Jarrow Formulas (green line).

The values (expressed in percentages) of DPPH reduction were normalised per tablet for all three pharmaceutical samples. All the tests were carried out in triplicate, and the results were reported as averages. It was noticed that DPPH scavenge depended on the CGA concentration in the samples (Figure 12).

The results obtained for the capacity to scavenge the DPPH radical, expressed in percentages, are given in Table 8.

It can be affirmed that CGA is active in all the three products, and is present in high concentrations, as mentioned by the producer on the label, and as such, has been determined through the analytical method employed in this study.

## 4. Conclusions

The screen-printed graphene-based electrode, modified with manganese phthalocyanine and with tyrosinase, was developed and used in researching and determining the electrochemical behaviour of chlorogenic acid. The applicability of the tyrosinase enzyme in obtaining enzymatic biosensors is of great importance in the field of medical research. These biosensors represent a promising technology for monitoring and detecting phenolic compounds due to advantages, such as high selectivity, low production costs, simple equipment, high response rate, and the potential for miniaturisation. From the obtained results, it can be stated that the manganese phthalocyanine facilitated the tyrosinase activity, being also a mediator of the electron transfer in the CGA oxidation process. By using cyclic voltammetry and square-wave voltammetry as detection methods, remarkable results were achieved—with applicability in laboratory practice. The calibration curve of GPH-MnPc-Tyr/SPE towards CGA showed linearity in the 0.1–10.48 µM concentration range and the values of LOD and LOQ were low, close to those obtained by other biosensors based on tyrosinase in the detection of phenolic compounds. Moreover, the biosensor was applied for the quantitative detection of CGA in three nutraceutical formulations, through cyclic voltammetry, employing the Folin–Ciocalteu spectrophotometric method for its validation, with similar results in both cases. Through the DPPH method, the antioxidant activity of the compound was also determined, thus demonstrating the antioxidant effect of CGA in the three nutraceutical products studied.

In conclusion, the new biosensor developed based on manganese phthalocyanine and tyrosinase can be used to detect the CGA content of pharmaceutical formulations, nutraceutical products, and food supplements. Some advantages, including reduced analysis time, small sample necessary, very good accuracy, and portability, render this electroanalytical method adequate for the quality control of these products.

## Figures and Tables

**Table 1:** Ipa vs. v^1/2^ linear regression equations, determination coefficients, active surface areas, geometrical areas, and the roughness factors of all the electrodes studied.

Electrode	Ipa vs. v^1/2^	R^2^	Active Area (cm^2^)	Geometrical Area (cm^2^)	Roughness Factor
GPH/SPE	I_pa_ (A) = 2.46 × 10^−4^ v^1/2^ (V·s^−1^)^1/2^ − 2.87 × 10^−5^	0.9949	0.1240	0.1256	0.9872
GPH-MnPc/SPE	I_pa_ (A) = 1.29 × 10^−4^ v^1/2^ (V·s^−1^)^1/2^ − 1.36 × 10^−5^	0.9953	0.1244		0.9904
GPH-MnPc-Tyr/SPE	I_pa_ (A) = 1.75 × 10^−4^ v^1/2^ (V·s^−1^)^1/2^ − 3.50 × 10^−5^	0.9956	0.1688		1.3439

**Table 2:** The values of the parameters were obtained from the cyclic voltammograms of all the electrodes immersed in 10^−3^ M CGA solution (the electrolyte support was 10^−1^ M PBS of pH = 7.0).

Sensor	Epa ^1^(V)	Ipa ^2^(µA)	Epc ^3^(V)	Ipc ^4^(µA)	Ipc/Ipa	ΔE ^5^(V)	E^1/2 6^
GPH-MnPc/SPE	0.190	4.30	0.117	−3.22	0.74	0.073	0.153
GPH-Tyr/SPE	0.283	7.65	0.095	−10.20	1.33	0.188	0.189
GPH-MnPc-Tyr/SPE	0.214	10.81	0.017	−14.14	1.31	0.197	0.115

^1^ Potential of the anodic peak; ^2^ Current of the anodic peak; ^3^ Potential of the cathodic peak; ^4^ Current of the cathodic peak; ^5^ ΔE = Epa − Epc; ^6^ E^1/2^ = (Epa + Epc)/2.

**Table 3:** The linear fitting equations (I_pc_ vs. v), R^2^, and Γ for both electrodes used in the electroanalysis.

Electrode	Equation	R^2^	Г (mol × cm^−2^)
GPH-MnPc/SPE	Ipc = 17.216 × 10^−5^ v (V·s^−1^) − 21.68 × 10^−5^	0.9895	3.34 × 10^−11^
GPH-MnPc-Tyr/SPE	Ipc = 77.199 × 10^−5^ v (V·s^−1^) − 91.531 × 10^−5^	0.9968	1.50 × 10^−10^

**Table 4:** Equation of linear dependence between Ipc and c, R^2^, LOD, and LOQ for GPH-MnPc-Tyr/SPE.

Electrode	Equation	R^2^	LOD (M)	LOQ (M)
GPH-MnPc-Tyr/SPE	Ipc = −0.0692 × −4.3141	0.996	1.40 × 10^−6^	4.69 × 10^−6^

**Table 5:** Sensitive materials, detection techniques, and limits of detection (LODs) of the main voltammetric sensors used for the detection of chlorogenic acid.

Sensitive Material	Detection Technique	LOD (µmol × L^−1^)	References
TAPB-DMTP-COFs/AuNPs ^1^	CV, DPV	9.5 × 10^−3^	[66]
LGN-MWCNTs-CuONPs-GCE ^2^	CV, DPV	1.25 × 10^−2^	[31]
MWCNTs/SPE ^3^	DPV	3.4 × 10^−1^	[24]
MIS/Au electrode ^4^	DPV	1.48 × 10^−1^	[67]
PASA/GCE ^5^	CV	0.4 × 10^−1^	[68]
Ir-BMI.PF_6_-PPO ^6^	SWV	9.1 × 10^−1^	[69]
IL/DMC/PE	SWV	1 × 10^−2^	[30]
Biosensor I and biosensor II ^7^	SWV	8 × 10^−1^ and 8.5 × 10^−1^	[70]
GPH-SPE ^8^ and GPH-GNP-SPE ^9^	CV	7.3 × 10^−1^ and 6.2 × 10^−1^	[28]
GPH-MnPc-Tyr/SPE	SWV	2.32 × 10^−1^	This study

DPV, differential pulse voltammetry; SWV, square-wave voltammetry; CV, cyclic voltammetry; ^1^ 1,3,5-tris(4-aminophenyl)benzene-2,5-dimethoxyterephaldehyde-covalent organic frameworks/gold nanoparticles; ^2^ lignin polymer-multi-walled carbon nanotubes-copper oxide nanoparticles-glassy carbon electrode; ^3^ multi-walled carbon nanotubes modified screen-printed electrode; ^4^ molecularly imprinted siloxane (MIS) film, prepared by sol–gel process, onto Au bare electrode; ^5^ poly (aminosulfonic acid) modified glassy carbon electrode; ^6^ 1-n-butyl-3-methylimidazolium hexafluorophosphate containing dispersed iridium nanoparticles (Ir-BMI.PF6) and polyphenol oxidase; ^7^ Biosensors based on bean sprout homogenate immobilised in chitosan microspheres (Biosensor I) and silica (Biosensor II) crosslinked with glutaraldehyde and epichlorohydrin; ^8^ screen-printed electrode modified with graphene; ^9^ screen-printed electrode modified with graphene and gold nanoparticles.

**Table 6:** Interference of chemically related compounds with the quantitative determination of CGA 10^−5^ M.

Interfering Compound	Concentration of the Interferents	Ratio	Recovery %	RSD %
-	-	-	100	0.50
L-ascorbic acid	10^−5^ M	1:1	97.45	1.82
Vanillic acid	10^−5^ M	1:1	99.15	0.60
Ferulic acid	10^−5^ M	1:1	97.24	1.97
p-Coumaric acid	10^−5^ M	1:1	96.81	2.28

**Table 7:** CGA concentrations in nutraceuticals obtained by the voltammetric method, and by Folin–Ciocalteu spectrophotometric method, respectively.

Food Supplement	Voltammetric Method C % CGA	Spectrophotometric Method C % CGA
Green Coffee Bean Complex (Adams Vision)	83.72	87.39
Green Coffee (Biotech USA)	79.77	80.21
Green Coffee Bean 400 mg Jarrow Formulas	79.72	80.11

**Table 8:** DPPH reduction capacity in the case of the three nutraceuticals.

Nutraceutical Product	Green Coffee Bean Complex Adams Vision	Green Coffee Biotech USA	Green Coffee Bean 400 mg Jarrow Formulas
% DPPH Reduction	91.66	91.05	93.07

## Data Availability

The authors confirm that the data supporting the findings of this study are available within the article.

## Supplementary Materials

The following supporting information can be downloaded at: https://www.mdpi.com/article/10.3390/ma15093221/s1, Figure S1: FTIR spectra for GPH-MnPc/SPE (red line) and for GPH-MnPc-Tyr/SPE (blue line), Figure S2: Effect of the amount of enzyme added on graphene and manganese phthalocyanine modified screen-printed carbon electrode on its sensitivity to catechol detection (10^−3^ M catechol, 10^−1^ M phosphate buffer solution as supporting electrolyte, at pH = 7.0). Applied potential −0.08 V vs Ag/AgCl., Figure S3: The effect of pH on tyrosinase activity, Figure S4: Cyclic voltammograms of the GPH/SPE (a), GPH-MnPc/SPE (c), GPH-MnPc-Tyr/SPE (e) immersed in 10^−3^ M catechol-10^−1^ M PBS solution registered with scan rates between 0.1–1.0 V × s^−1^. Plot of Ipa vs. v^1/2^ in the case of GPH/SPE (b), GPH-MnPc/SPE (d), GPH-MnPc-Tyr/SPE (f), Table S1: The values of the parameters obtained from the cyclic voltammograms of all the electrodes immersed in 10^−3^ M catechol-10^−1^ M PBS double solution.
