# Supplementary material for: Tyrosinase-Based Biosensor—A New Tool for Chlorogenic Acid Detection in Nutraceutical Formulations
Source: Materials (Basel). 2022 Apr 29;15(9):3221. doi: 10.3390/ma15093221 (PMC9104151; doi:10.3390/ma15093221)
Supplement: Supplementary file 1 [file materials-15-03221-s001.zip › materials-1628619-supplementary.pdf]

Materials

Supplementary Material

Tyrosinase-Based Biosensor—A New Tool for Chlorogenic Acid Detection in Nutraceutical Formulations

Irina Georgiana Munteanu and Constantin Apetrei *

Department of Chemistry, Physics and Environment, Faculty of Sciences and Environment, Dunărea de Jos University of Galați, 47 Domneasca Street, 800008 Galați, Romania; georgiana.munteanu@ugal.ro
* Correspondence: apetreic@ugal.ro; Tel.: +40-727-580-914

Supplementary information

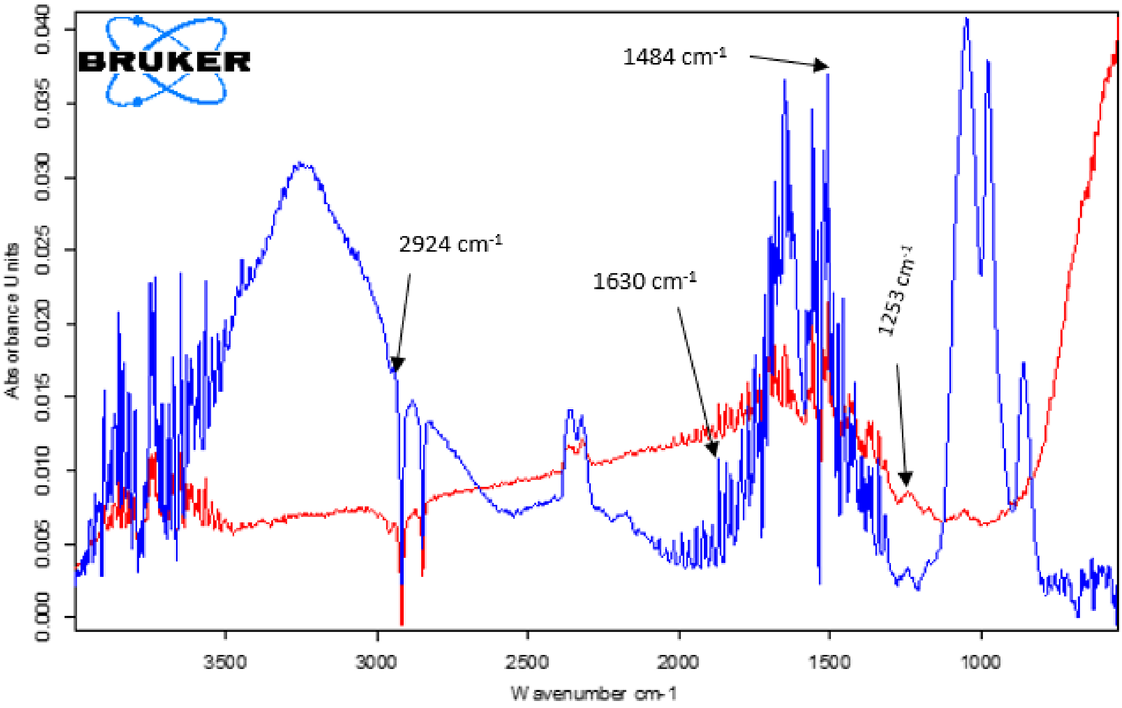

Figure S1. FTIR spectra for GPH-MnPc/SPE (red line) and for GPH-MnPc-Tyr/SPE (blue line).

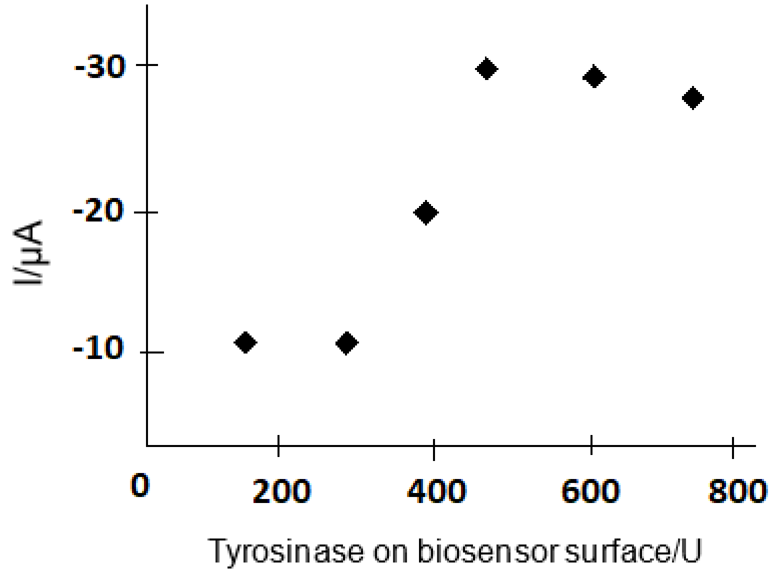

Figure S2. Effect of the amount of enzyme added on graphene and manganese phthalocyanine modified screen-printed carbon electrode on its sensitivity to catechol detection (10^{-3}M catechol, phosphate buffer solution 10^{-1} M as supporting electrolyte, at pH=7.0). Applied potential -0.08 V vs Ag/AgCl.

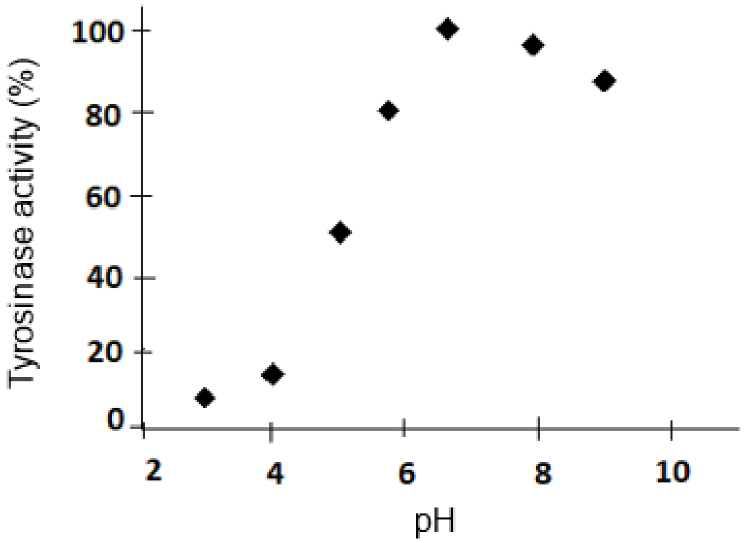

Figure S3. The effect of pH on tyrosinase activity.

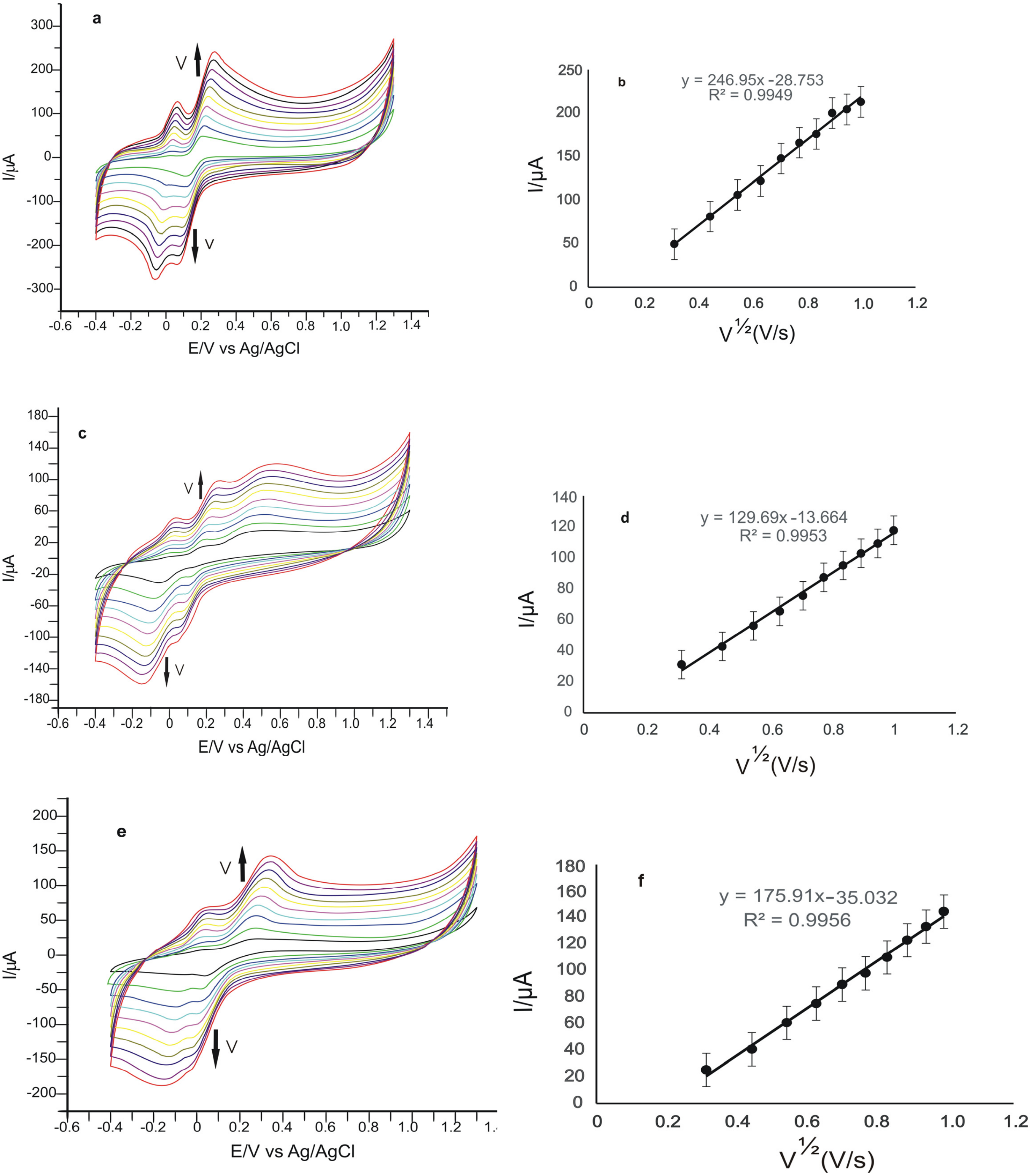

Figure S4. Cyclic voltammograms of the GPH/SPE (**a**), GPH-MnPc/SPE (**c**), GPH-MnPc-Tyr/SPE (**e**) immersed in 10^{-3} M catechol – 10^{-1} M PBS solution registered with scan rates between 0.1-1.0 V x s^{-1}. Plot of Ipa vs $v^{1/2}$ in the case of GPH/SPE (**b**), GPH-MnPc/SPE (**d**), GPH-MnPc-Tyr/SPE (**f**).

Table S1. The values of the parameters obtained from the cyclic voltammograms of all the electrodes immersed in 10^{-3} M catecol – 10^{-1} M PBS double solution. [1] Current of the anodic peak; [2] Current of the cathodic peak; [3] Potential of the anodic peak; [4] Potential of the cathodic peak; [5] Half-wave potential; [6] ΔE= Epa – Epc.

Sensor	Ipa[1] (μA)	Ipc[2] (μA)	Ipc/Ipa	Epa[3] (V)	Epc[4] (V)	$E_{1/2}$ [5] (V)	ΔE[6] (V)
GPH-SPE	49.50	-42.55	0.85	0.204	0.117	0.160	0.087
GPH-MnPc/SPE	32.02	-30.69	0.95	0.447	-0.055	0.251	0.392
GPH-MnPc-Tyr/SPE	25.57	-32.65	1.27	0.231	-0.077	0.154	0.154
